# Race Differences in Patterns and Consequences of Mothers’ Advice to Midlife Children

**DOI:** 10.1093/geroni/igaf122.1752

**Published:** 2025-12-31

**Authors:** Robert Frase, J Jill Suitor, Megan Gilligan, Destiny Ogle, Ranran He, Di Wang

**Affiliations:** Southern Illinois University, Carbondale, Illinois, United States; Purdue University, West Lafayette, Indiana, United States; University of Missouri, Columbia, Missouri, United States; Purdue University, West Lafayette, Indiana, United States; Purdue University, West Lafayette, Indiana, United States; Purdue University, West Lafayette, Indiana, United States

## Abstract

Although advice is often conceptualized as a positive dimension of intergenerational support, a growing body of research has examined the potentially adverse effects of advice from older parents on adult children’s well-being. However, no attention has been given to exploring race differences in this association. Drawing from theories of intergenerational solidarity and using data from 670 midlife children nested in 276 later-life families who participated in wave 2 of the Within-Family Differences Study, we extend this scholarship by examining whether the association between advice from mothers and adult children’s depressive symptoms differs for Black and White adult children. On one hand, advice is a potential source of interpersonal tension, and some research indicates that negative facets of family relationships (e.g., arguments, disappointment) have stronger adverse effects on the well-being of Black than White adults, suggesting that the association between receiving advice from mothers and adult children’s depressive symptoms would be amplified among Black adults. On the other hand, research indicating higher levels of intergenerational solidarity and more frequent exchanges of support in Black than White families would suggest that Black adult children would be more accepting of advice from mothers, attenuating the effects of advice on Black children’s well-being. Findings from multilevel models revealed that the association between receiving advice from older mothers and depressive symptoms was stronger among Black than White midlife children. Our results both augment existing scholarship on the potentially harmful effects of advice on well-being and underscore the importance of considering social-structural characteristics in this research.

